# Clinical Manifestations and Distribution of Cutaneous Leishmaniasis in Pakistan

**DOI:** 10.1155/2011/359145

**Published:** 2011-11-22

**Authors:** Abaseen Khan Afghan, Masoom Kassi, Pashtoon Murtaza Kasi, Adil Ayub, Niamatullah Kakar, Shah Muhammad Marri

**Affiliations:** Department of Pathology, Bolan Medical College, 8-13/36 Kasi Road, Balochistan, Quetta 87300, Pakistan

## Abstract

Cutaneous leishmaniasis (CL) is a rising epidemic in Pakistan. It is a major public health problem in the country especially alongside regions bordering the neighboring Afghanistan and cities that have had the maximum influx of refugees. The purpose of our paper is to highlight the diverse clinical manifestations of the disease seen along with the geographic areas affected, where the hosts are particularly susceptible. This would also be helpful in presenting the broad spectrum of the disease for training of health care workers and help in surveillance of CL in the region. The increased clinical diversity and the spectrum of phenotypic manifestations noted underscore the fact that the diagnosis of CL should be not only considered when dealing with common skin lesions, but also highly suspected by dermatologists and even primary care physicians even when encountering uncommon pathologies. Hence, we would strongly advocate that since most of these patients present to local health care centers and hospitals, primary care practitioners and even lady health workers (LHWs) should be trained in identification of at least the common presentations of CL.

## 1. Background

Cutaneous leishmaniasis (CL) is a rising epidemic in Pakistan [1]. It is a major public health problem in the country especially alongside regions bordering the neighboring Afghanistan and cities that have had the maximum influx of refugees [2]. Pakistan in particular, as highlighted by Postigo, has been a focus of both anthroponotic cutaneous leishmaniasis caused by *Leishmania tropica (L*. *tropica*) and zoonotic CL caused by *Leishmania major (L*. *major*) with epidemics occurring in various parts of the country [3]. *L. tropica* is mostly seen in urban areas whereas *L. major* is more common in rural areas of the country [4]. 

The purpose of our paper is to highlight the diverse clinical manifestations of the disease seen along with the geographic areas affected where the hosts are particularly susceptible. This would also be helpful in presenting the broad spectrum of the disease for training of health care workers and help in surveillance of CL in the region [3].

## 2. Methodology for Our Review

We searched the terms “cutaneous”, “leishmaniasis,” and “Pakistan,” in Pubmed which retrieved a total of 67 articles. These articles were then systematically reviewed with focus on the number of people affected in different areas of the country and the clinical manifestations of the disease documented by the authors. A summary of the studies selected is outlined in Table 1. References citing other studies done in the region were then also studied for the aforementioned objectives. 

## 3. Clinical Manifestations of Cutaneous Leishmaniasis

**Figure 1:**
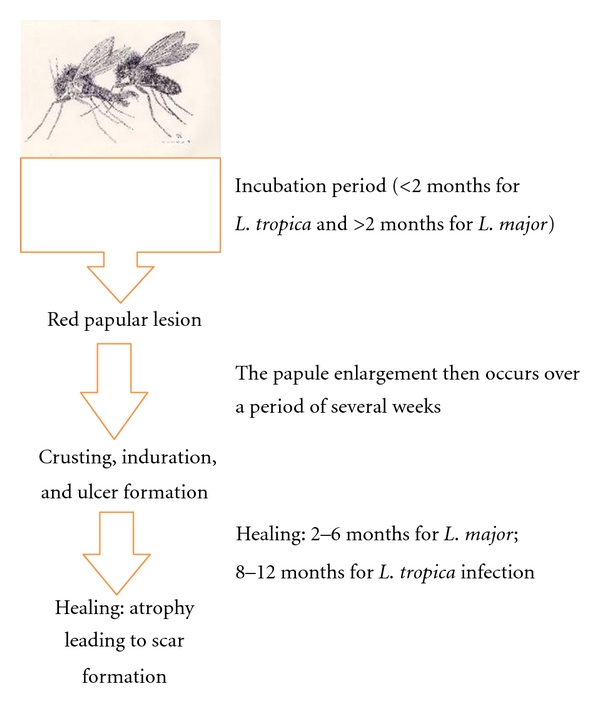
Typical sequence of events leading to the formation of the typical oriental or “yearly sore” called “kal dana.” Description adopted from excellent review by Arfan u Bari et al., 2009. Picture of Sandflies obtain through the courtesy of Bruce Alexander, Research Fellow in Molecular and Biochemical Parasitology Group, Liverpool School of Tropical Medicine.

**Figure 2:**
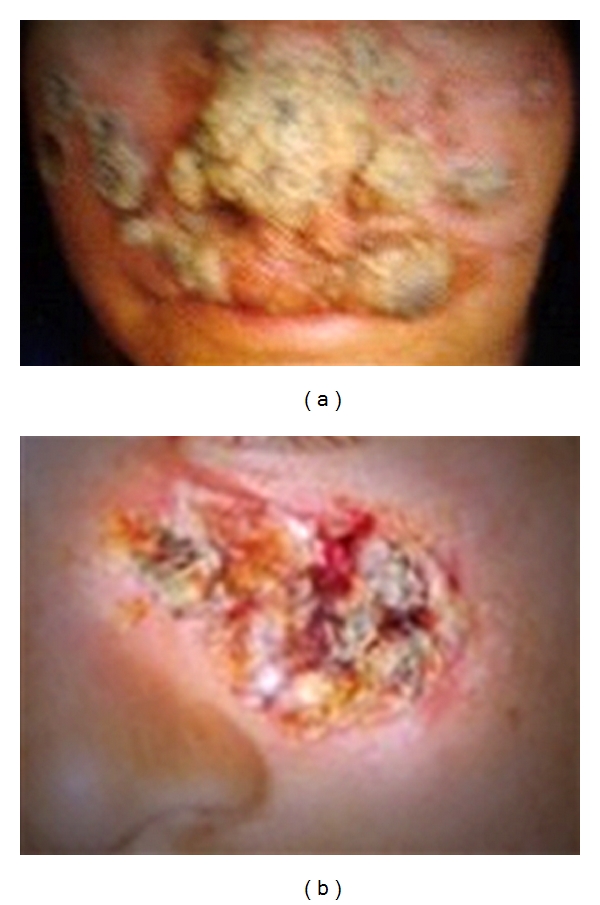
Disfiguring nature of lesions of CL on exposed parts of the body, particularly the face (copyright Kassi et al. [2]). Permission taken under creative commons attribution license.

CL is usually noted on exposed parts of the body, mainly arms, face, and legs. The clinical manifestations are extremely diverse including unusual sites and atypical morphologies. Typically, the natural course of the lesions seen in CL is outlined by Figure 1. The lesions typically are not painful, but are associated with significant stigma associated with the disease (Figure 2). Women and children are particularly affected. As noted, however, it is increasingly seen in various unusual forms, for example, as fissures on lips, with lupoid features on face and/or psoriasiform plaques on nose [5, 6]. The myriad of manifestations seen is an outcome of the interplay between the parasite infection and host's immune responses [4]. “On one end of the spectrum of CL is the classical oriental sore in which spontaneous cure and immunity to reinfection is the result of an effective parasiticidal mechanism. On the other end of the spectrum is diffuse cutaneous leishmaniasis in which metastatic cutaneous lesions develop and the patient rarely, if at all, spontaneously develops immunity to the parasite” [7]. 

## 4. Causative Agent in Pakistan

The main causative agent noted in our review of the studies was L. *major* followed by *L*. *tropica*. This was also highlighted in a review on molecular epidemiology of leishmaniasis in Asia, where in southern dry areas of the country, *L*. major was seen more often [28]. However, as noted by Katakura as well as Marco et al., there was no clear association between the skin lesions and the type of leishmaniasis, probably referring to host factors and immunoinflammatory responses being more important in determining the type and severity of lesions noted [28–31]. Likewise, no correlation between the type of CL lesions and the causal *leishmania* parasites was noted in a gene sequencing study on samples from both provinces of Sindh and Balochistan [14]. However, as outlined in Table 1, in the same study, there was indeed an association between the type of *leishmania* parasite and the altitude of the region, with *L. major* (97.9%) being the predominant type of parasite in lowland areas, while *L. tropica* (76.2%) was more common in highland areas [14]. *L. tropica* was also the strain most commonly found in a study from Multan and another study with a small sample size from various parts of the country [32, 33]. Clinical presentations appear to vary by endemic regions [31]. 

## 5. Distribution of Cutaneous Leishmaniasis in Pakistan

**Figure 3:**
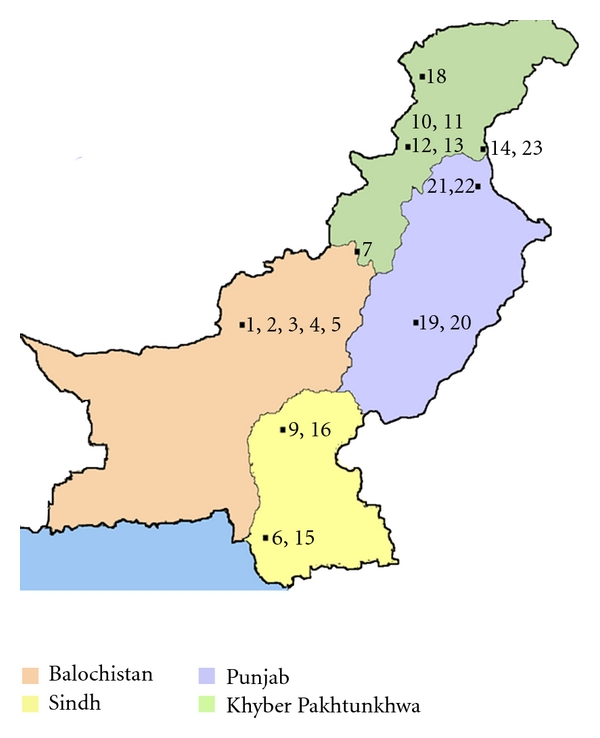
Distribution of CL in the 4 provinces of Pakistan (numbers represent the studies as noted in the references). As noted in the map as well, the province of Balochistan followed by Khyber Pakhtunkhwa appears to have taken a major toll. Most of the cities and hospitals where the disease has been identified serve as major tertiary care referral centers for the rest of the province. The exact estimates in adjoining cities and rural areas are underestimated and not well known.

The country Pakistan is divided into 4 provinces: Punjab, Sindh, Khyber Pakhtunkhwa, and Balochistan along with Azad Jammu Kashmir (AJK). As noted from the research studies cited in Table 1, some parts of the country are more affected than others (Figure 3). 

 Balochistan, the largest province by area located in the southwest of Pakistan, appears to have taken a significant toll followed by Khyber Pakhtunkhwa [9]. The geographic distribution, as noted by Firdous et al., is a function of the sandfly vector compounded with activities disturbing their habitat including wars as well as deforestation and agricultural activities [4, 34]. Breeding of animals is also thought to play a contributory role, along with “dark niches and cracks in the ground providing suitable habitat for the sandflies” [24, 28]. Within Balochistan, areas where most patients have been reported from are Quetta, Ormara, and Uthal [35]. Quetta is a metropolitan city and the capital of the province. Reasons for more cases of CL being reported from the capital of the province are that people belonging to different castes live there along with many refugees who were from the adjacent war-torn country of Afghanistan and migrated during the early 1980s and 1990s [1]. The hospitals and clinics serve as major teaching/tertiary care centers not only for the entire province but also for neighboring adjoining areas of Afghanistan.

The increased incidence noted in some parts of the country where major teaching hospitals and where most of the research studies are conducted may be a function of better awareness and more testing of chronic skin lesions for CL. Since a lot of these hospitals and research centers cater and serve as major referral centers for entire provinces in some cases, the true incidence especially in outskirts and rural areas is currently not exactly known and may be an underestimate [11]. For example, even in the survey-based study of thousands of school children, predominant population affected were male children; the severity and the proportion of women affected may not be entirely known given sociocultural factors [9]. 

## 6. The Road Ahead

The increased clinical diversity and the spectrum of phenotypic manifestations underscore not only fact that the diagnosis of CL should be not only considered when dealing with common skin lesions, but also highly suspected by dermatologists and even primary care physicians even when encountering uncommon pathologies [26]. Diagnosis in these atypical cases, which are now more frequently being seen, can particularly be challenging. Here, we would strongly advocate that since most of these patients present to local health care centers and hospitals, primary care practitioners and even lady health workers (LHWs) should be trained in identification of at least the common presentations of CL, since the majority of patients especially in rural areas do not have access to dermatologists [36]. Providing training to LHWs in identification of CL can prove helpful not only for women to whom they have access to but also in developing better surveillance systems of catching sporadic epidemics early. 

A lot of research is being conducted by the efforts of local universities themselves and some interventions by NGOs and UNHCR. As noted by Brooker and colleagues as well, further work and interventions within the country of Pakistan would have to be supported by the Ministry of Health for improving diagnostic abilities and implementing preventive and treatment programs in more regions of the country before the epidemic continues to rise further [13].

## 7. Conclusions

- CL is usually seen in exposed parts of the body.
- The clinical manifestations are extremely diverse including unusual sites and atypical morphologies.
- The myriad of manifestations seen is an outcome of the interplay between the parasite infection and host's immune responses.
- The increased clinical diversity and the spectrum of phenotypic manifestations underscore not only fact that the diagnosis of CL should be not only considered when dealing with common skin lesions, but also highly suspected by dermatologists and even primary care physicians even when encountering uncommon pathologies.
- Hence, we would strongly advocate that since most of these patients present to local health care centers and hospitals, primary care practitioners and even Lady Health Workers (LHWs) should be trained in identification of at least the common presentations of CL, since the majority of patients especially in rural areas do not have access to dermatologists.

## Figures and Tables

**Table 1:** Summary of studies in Pakistan on clinical manifestations of Cutaneous Leishmaniasis alongside their geographic distribution.^†^

		Period	City/province	Number of cases	Method of diagnosis	Species of *Leishmania *	Type of lesions seen
(1)	Gazozai et al. [8]	2005–2007	Quetta, Balochistan	300	Histopathological examination; skin smears		Nodules, plaques, ulcers and/or scarring

(2)	Firdous et al. [4]	2005–2007	Quetta, Balochistan; adjoining areas noted included Sibi, Zhob, Loralai, Pishin, and Kohlu	207	Histopathological examination	L. major	94% of lesions on upper and lower extremities in military personnel. 77 (37%) had a single lesion, 46 (19%) had two lesions, 19 (9%) had three lesions, and 35% had four lesions. The lesions were mostly noduloulcerative plaques with or without crusting

(3)	Kakarsulemankhel et al. [9]	1996–2001	Data from 7 zones of the province of Balochistan	School children: 17–22 years: 1617 cases 11–16 years: 2643 cases 5–10 years: 3210 cases 8,007 patients with active lesions presenting to hospitals/clinics.	Survey data, clinical and/or histopathological examination employed in different regions		Dry lesions more common in Quetta; wet lesions in other 6 regions of the province. Both active lesions and scarring were noted

(4)	Raja et al. [10]	1998	Balochistan	1709 patients; 2% (37) had unusual presentations	Clinical and histopathology		These included acute paronychial, chancriform, annular, palmoplantar, zosteriform, and erysipeloid forms in a total of 37 patients

(5)	Kassi et al. [1, 11]		Quetta, Balochistan	166	FNAC/Histopathology		Dry ulcerated lesions were noted to be more common on face, arms, and legs

(6)	Shoai et al. [12]	1997–2001	Karachi, Sindh (areas of origin of patients were noted from all 4 provinces, mainly from Sindh (40.5%) and Balochistan (28%))	175	Histopathological examination and PCR	Both L. Tropica and L. major	h 60 (82.6%) showed wet type of lesions characterized by exudates, redness, and inflamed margins. The remaining 15 (17.3%) were of dry and nodular type covered by crust

(7)	Brooker et al. [13]	2002-2003	19 neighboring villages in Balochistan and Khyber Pakhtunkhwa	7,305 persons	Clinical diagnosis		Overall, 650 persons (2.3%) had anthroponotic CL (ACL) lesions only, 1,236 (4.4%) had ACL scars only, and 38 persons had both ACL lesions and scars

(8)	Myint et al. [14]	2008	Samples from both Sindh and Balochistan: 48 cases from lowland areas; 21 cases from highland areas	69	Gene sequencing	47 L. Major and 1 L. Tropica in lowland areas. 5 L. Major and 16 L. Tropica in highland areas.	Again, no correlation between clinical presentation (wet, dry and/or mixed types of cutaneous lesions) and causal leishmania parasites

(9)	Bhutto et al. [15]	1996–2001	Jacobabad, Larkana, and Dadu districts of Sindh province and residents of Balochistan province	1210	Clinical; a giemsa-stained smear test and histopathology		Clinically, the disease was classified as dry papular type, 407 cases; dry ulcerative type, 335 cases; wet ulcerative type, 18 cases

(10)	Bari et al. [7]	2009	Peshawar, Khyber Pakhtunkhwa	2	Slit skin smear and FNAC		Cutaneous fissures on lip and dorsum of finger

(11)	Rahman et al. [5]	2006–2008	Peshawar, Khyber Pakhtunkhwa	1680 498 patients from different areas of Peshawar; 688 from FATA; 89 from other urban and rural areas of the province	Skin smear for LD bodies		Typical “oriental sore” noted in 1512 cases; 168 had an atypical presentation. Several chronic nonhealing ulcers were noted.

(12)	Ul Bari and Ejaz [6]	2009	Peshawar, Khyber Pakhtunkhwa	1	Skin smear preparation		Rhinophyma-like plaque on nose

(13)	Ul Bari [16]	2009	Peshawar, Khyber Pakhtunkhwa	72	Smear preparations/histopathological examination		Nasal leishmaniasis. Main morphological patterns included psoriasiform (30), furunculoid (8), nodular (13), lupoid (8), mucocutaneous (4), and rhinophymous (3)

(14)	Qureshi et al. [17]	2007	Abbottabad, Khyber Pakhtunkhwa	1	Histopathology		Typical butterfly-like rash seen in SLE

(15)	Saleem et al. [18]	2004–2006	Karachi, Sindh	100	Clinical and histopathological examination		Nodules, plaques, ulcers, crusted ulcers, lupoid lesions, and plaques with scarring were mainly noted

(16)	Bhutto et al. [19]	2009	Larkana, Sindh	108	Polymerase chain reaction (PCR)	L. Major (105) L. Tropica (3)	

(17)	Ul Bari and Ber Rahman [20]	2004–2006	Punjab and Khyber Pakhtunkhwa	60	Slit-skin smear and histopathology		Presentation either (a) wet type (early ulcerative, rural) or (b) dry type (late ulcerative, urban)

(18)	Rowland et al. [21]	1997	Timergara, Dir, Khyber Pakhtunkhwa	9200 inhabitants	Clinical diagnosis; sample of cases confirmed with microscopy and PCR	Possible L. tropica based on Noyes et al. [22]	38% of the 9200 inhabitants bore active lesions, and a further 13% had scars from earlier attacks

(19)	Mujtaba and Khalid [23]	1995–1997	Multan, Punjab	305	Giemsa-stained smear from the lesion		All the lesions were of the dry type. Most of the lesions (97%) were present on exposed areas of the body

(20)	Ayub et al. [24]	1999–2000	Multan, Punjab	173	Smear for LD bodies		Clinically all the lesions were of dry type, with 67% present on legs

(21)	Anwar et al. [25]	2004	Khushab district, Punjab	105	FNAC of the lesion for first 4 cases; only history and clinical assessment for remaining		Disseminated forms noted in multiple cases; with 1 patient with more than 50 lesions

(22)	Bari and Rahman [26]	2002–2006	Rawalpindi, Sargodha, and Muzaffarabad	718 patients with CL; study was on 41 patients with unusual presentations	Clinical and histopathological examination		Common unusual presentations noted were lupoid leishmaniasis in 14 (34.1%), followed by sporotrichoid 5 (12.1%), paronychial 3 (7.3%), lid leishmaniasis 2 (4.9%), psoriasiform 2 (4.9%), mycetoma-like 2 (4.9%), erysipeloid 2 (4.9%), and chancriform 2 (4.9%)

(23)	Ul Bari and Raza [27]	2006–2008	Muzaffarabad, Azad Jammu and Kashmir	16	Histopathological examination		Cutaneous lesions resembling lupus vulgaris or lupus erythematosus, mainly over face. Morphological patterns included erythematous/infiltrated, psoriasiform, ulcerated/crusted, and discoid lupus erythematosus

^†^As noted, the province of Balochistan followed by Khyber Pakhtunkhwa appears to have taken a major toll. Most of the cities and hospitals where the disease has been identified serve as major tertiary care referral centers for the rest of the province. The exact estimates in adjoining cities and rural areas are underestimated and not well known.
